# Effect Analysis of Soil Type and Silt Content on Silt-Based Foamed Concrete with Different Density

**DOI:** 10.3390/ma13173866

**Published:** 2020-09-01

**Authors:** Hongbo Zhang, Xiaolin Qi, Chuanyi Ma, Jianqing Wu, Yufeng Bi, Renjuan Sun, Jintao Yu, Dongdong Xie, Jie Song

**Affiliations:** 1 School of Qilu Transportation, Shandong University, Jinan 250061, China; zhanghongbo@sdu.edu.cn (H.Z.); qixiaolinsdu@163.com (X.Q.); yujintaoheart@163.com (J.Y.); 2 Shandong High-Speed Group Co. Ltd., Jinan 250002, China; machuanyi2006@163.com (C.M.); m13082918362@163.com (D.X.); 3 Suzhou Research Institute, Shandong University, Suzhou 215123, China; 4 Shandong Provincial Communications Planning and Design Institute Co. Ltd., Jinan 250031, China; biyf@163.com (Y.B.); tom1442@sina.com (J.S.)

**Keywords:** foamed concrete, silt, coarse particle content, mechanical properties, air-void structure

## Abstract

This paper investigates the influence of silt content and coarse particle content on the mechanical and physical properties of foamed concrete, including compressive and flexural strength, modulus of elasticity, water absorption, drying shrinkage, and air-void structure. Four types of silt with different coarse particle contents were obtained by soil mixing. The results showed that high density, low silt content, and high coarse particle content can provide better mechanical properties. High silt content and high coarse particle content would lead to lower drying shrinkage. Silt content was the main factor affecting the pore distribution of foamed concrete, and higher coarse particle content could optimize the air-void structure. Meanwhile, the change in air-void structure can accordingly affect the water absorption of foamed concrete. Results showed that, at the same density and silt content, higher coarse particle content can optimize the physical and mechanical properties of foamed concrete.

## 1. Introduction

Foamed concrete refers to a type of lightweight material formed by mixing cement-based cementitious material, water, foam, and aggregate according to a certain proportion. It has the characteristics of light weight (density of 400–1850 kg/m^3^), high strength, excellent acoustic and thermal isolation, high resistance to fire, lower cost of raw materials, high flowability, and easier pumping and application [1,2,3]. Although foamed concrete is still not proclaimed in the construction industry, it already has non-structural and structural applications, even though the latter are still under investigation. Currently used foamed concrete is mainly divided into four categories. Firstly, it is used as non-structural filling material in ground works, because of its flowability features, higher strength, and thermal properties. It is a superlative material for voids such as old sewers, storage tanks, basements, piping, and voids under roadways [4]. In addition, it is also used as subgrade stabilizing material for its relatively higher strength [5] and light weight [6]. Thirdly, it is used as non-structural element of sandwich wall and slab elements, where the light weight and thermal insulation properties of foamed concrete are also used [7]. At last, foamed concrete can also be combined with steel reinforcement as the main structural element [8,9].

Due to the wide distribution of soft soil foundation bridge jumping and uneven settlement are common, thus affecting the driving safety of highways. Engineering practice proved that the foam concrete subgrade has good long-term dynamic stability [10]. The use of foamed concrete for subgrade filling can effectively reduce the additional stress of foundation, thus effectively controlling the settlement of subgrade without soft soil foundation treatment. Foamed concrete has good integrity and small dead weight, and the method of choosing foam concrete as subgrade filler achieved good results in controlling settlement of soft soil subgrade [11], while it can also reduce the cost of follow-up operation and maintenance, with significant comprehensive benefits [12].

Although the application effect of foamed concrete in subgrade filling is excellent, it is not currently widely promoted and applied. The main reason is that foamed concrete needs a lot of cement. Furthermore, the use of cement increases the cost of producing foamed concrete, making it still too expensive to produce foamed concrete relative to common subgrade fillers such as soil and gravel. According to our previous research, the capacity of cement foamed concrete is excessive compared to soil foundation. At the same time, the production, preparation, and use of cement will cause serious dust pollution and damage to the environment [13]. However, for foamed concrete, the existence of cement as a cementitious material is indispensable. To further promote lightweight materials, it is necessary to add a large amount of cheap aggregates in foamed concrete to reduce the use of cement, thus reducing the cost and protecting the environment. For example, a study undertaken by Kearsley and Wainwright [14] showed that up to 67% of the cement could be replaced with fly ash without any significant reduction in strength.

Aggregate is one of the main constituent materials of foamed concrete, which plays the role of skeleton support in the foamed concrete structure, reducing the volume change caused by dry shrinkage and wet swelling of cementitious materials in the process of setting and hardening [15]. As the main support for the internal structure of foamed concrete, aggregate can greatly impact its mechanical properties. The fineness of particles has a significant effect on the strength of foamed concrete. Results showed that foamed concrete mixed with fine sand would result in greater compressive strength than that of coarse sand at various densities [16]. Therefore, in order to meet the requirements of working performance and strength, soil-based foamed concrete can be prepared using of a type of soil containing both coarse particles and fine particles. Song et al. [17] revealed the basic soil characteristics of silt, such as uniform particle size, high roundness of particles, and strong hydrophilicity. In the research of Zhang et al. [18], silt could be used to replace the amount of cement to prepare foamed concrete with well-balanced properties. However, silt has a wide distribution area and different types, and the composition ratio of coarse particles and fine particles is different. The effects of different particle size distribution on the properties of foamed concrete are unknown.

According to Test Methods of Soils for Highway Engineering [19], when the content of coarse particles in the soil is greater than 25%, the coarse grains can play the role of skeleton in the soil and have considerable influence on the soil characteristics. Four coarse particle contents, 25%, 30%, 35%, and 40%, were selected for this test. This paper investigates the effects of the silt coarse particle content, silt content, and wet density on the physical and mechanical properties of silt-based foamed concrete, such as water absorption, drying shrinkage, SEM (scanning electron microscopy), compressive strength, flexural strength, and elastic modulus. These studies provide a new theoretical basis for its use as road material and promote the application of silt-based foamed concrete in engineering.

## 2. Materials and Methods

### 2.1. Materials

Ordinary P.O.42.5 Portland cement (Shuangfengshang Cement Company, Zibo, China) was adopted. Physical properties of the cement and the chemical composition of the cement are listed in Table 1 and Table 2.

The raw materials used in this experiment were silt and clayey silt from Dongying, China, and the experimental soil was made by mixing the two raw materials in a certain proportion. Some other parameters are shown in Table 3.

**Figure 1:**
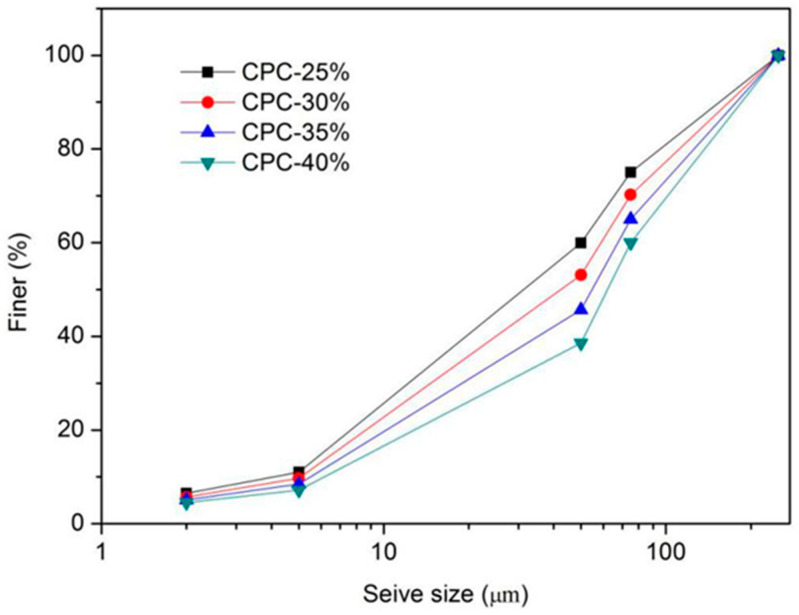
Particle size distribution curves.

Coarse particle content (CPC) refers to the ratio of the soil particle mass with particle size greater than 0.075 mm to the total mass of the soil sample. Four kinds of soil samples with coarse particle contents of 25%, 30%, 35%, and 40% were studied, and the particle size distribution curves of the four different coarse grain contents are shown in Figure 1 and Table 4.

The foaming agent used in the test was a protein-based anionic foaming agent produced by Chilong Building Energy Saving Technology Co. Ltd. (Shandong, China). The dilution ratio was 35–45 times and the foaming ratio was 800–1000 times.

### 2.2. Mix Proportions of Foamed Concrete

The mix proportions with three different series of density, silt content, and coarse particle content are shown in the Table 5. The effects of different silt types and content on the properties of foamed concrete were studied by means of fixing the water-to-solid ratio. The water-to-solid ratio (W/S) is defined as the ratio of water consumption to the sum of cement and silt consumption per cubic meter. The water-to-solid ratio (W/S) is one of the key factors affecting the properties and performance of foamed concrete; according to the relevant requirements [20], the water-to-solid ratio (W/S) should be selected between 0.55 and 0.65. With the decrease in W/S, the structure becomes more compact and complete. In order to ensure higher performance, the water-to-solid ratio (W/S) was 0.55 for all the specimens.

In Table 5, Series I aimed to investigate the influence of wet density on the performance of foamed concrete by changing wet density from 600 kg/m^3^ to 800 kg/m^3^. The silt content (SC) was 20% and coarse particle content (CSC) was 25%, where SC is the mass of the silt divided by the total mass of the solid. Series II was used to study the influence of silt content on the performance of foamed concrete. The silt content ranged from 0 to 40%. The wet density was 700 kg/m^3^ and the coarse particle content was 25%. As seen in the Table 5, Series III was set to explore the influence of different types of silt on foamed concrete. Keeping silt content at 40%, the four types of silt corresponded to the four coarse particle contents of 25%, 30%, 35%, and 40%.

### 2.3. Specimen Preparation

**Figure 2:**
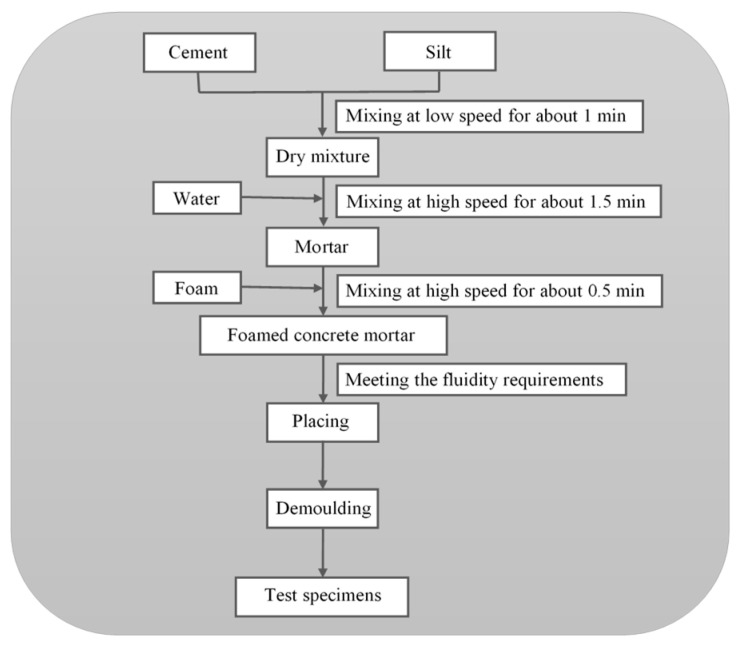
Procedure of preparation of foamed concrete.

The sample preparation process is shown in Figure 2. Cement mortar was obtained by mixing raw materials. Foamed concrete slurry was produced by adding preformed foam (Chilong Construction Material Company, Yantai, China) into cement mortar. The flow value should be measured before pouring the mortar into the mold. A clean square glass plate (500 mm × 500 mm) and an open-ended cylinder (with diameter and length of 80 mm) were prepared for the fluidity test [21]. Fresh foamed concrete was poured into the cylinder on the top of the glass plate until the cylinder was full. Then, the cylinder was lifted vertically, and the fresh foamed concrete spread on the glass plate. The fluidity of the mixture was controlled at 180 ± 20 mm [22]. The flow value of the mortar was as shown in Table 5. It can be seen that the flow value increased with the increase in density, silt content, and coarse particle content, all within the limits. Wet density was calculated from the weight and known volume of the standard vessels. The specimens could be casted after meeting the requirements of wet density and flow value. Test specimens were cured at 95% ± 3% relative humidity and 22 ± 2 °C until testing.

### 2.4. Testing Methods

Compressive strength, flexural strength, and elastic modulus were measured following the Chinese GB/T 11969-2008 standard [23]. According to Chinese standard, the size of the test specimens for compressive strength was 100 mm × 100 mm × 100 mm. At least three specimens of each mix should be measured. The loading rate was controlled as 2 kN/s. The flexural strength of foamed concrete was tested under the four-point bending method at the age of 28 days. The electronic universal testing machine (Shijin Testing Machine Company, Jinan, China) was used to apply a vertical load on the test specimen (100 mm × 100 mm × 400 mm) with a loading rate 0.2 kN/s. The elastic modulus was measured using 100 mm × 100 mm × 300 mm specimens and a loading rate of 0.1 kN/s.

According to the Chinese JG/T266-2011 [24], a cube with a size of 100 mm × 100 mm × 100 mm was used to test the water absorption of foamed concrete. Firstly, these specimens were immersed in water, while the surrounding environment remained consistent with the curing conditions until these specimens reached a constant weight. After weighing, these specimens were dried at 110 °C until reaching a consistent mass, after which the dry weight was obtained.

A rectangular specimen with a size of 40 mm × 40 mm × 160 mm was selected to conduct the drying shrinkage test [23]. In the center of the two undersurfaces of the specimen, a hole with a diameter of 6–10 mm and a depth of 13 mm was drilled in each. Sodium silicate cement slurry (Hengli Chemical Company, Tongxiang, China) was poured into the hole, and then the shrinkage head was buried. The central line of the shrinkage head coincided with the central line of the specimen, and the underside of the specimen was flat. After the specimen was placed for one day, it was immersed in a constant-temperature tank (Jinghong Testing Machine Company, Shanghai, China) with a water temperature of 20 ± 2 °C. The water surface was 30 mm above the specimen and maintained for 72 h. Then, the specimen was removed from the water and wiped with a wet cloth. The shrinkage head was wiped clean and the specimen was weighed immediately. The origin of the instrument was adjusted with a standard bar, and then the initial length of the specimen was measured immediately according to the indicated test direction, while taking the initial dial simultaneously. The specimen was placed in a temperature- and humidity-regulating chamber with a temperature of 20 ± 2 °C and a relative humidity of 43% ± 2%. In the first five days of the test, the specimens were measured in a room of 20 ± 2 °C once a day, and then every four days until the mass change was less than 0.1%. At last, the specimen was oven-dried (Jiangdong Machine Company, Suzhou, China) to a constant mass, which was recorded as the dry mass.

The 28-day air-void structure was investigated using scanning electron microscopy (SEM). The instrument used in this experiment was the S-4800 ultra-high-resolution scanning electron microscope produced by Toshiba in Japan, which uses a magnification ratio of 30×. Three images covering an area of 38.7 mm^2^ were analyzed for each mix. After the images were captured, Image Pro Plus 6.0 image processing software (EPIX Inc., Buffalo Grove, IL, USA) was adopted to analyze and process the microscopic images. The pore size, area, and other parameters of each pore were calculated through the software.

## 3. Test Results and Discussion

### 3.1. Effect of Density on the Properties of Foamed Concrete

**Figure 3:**
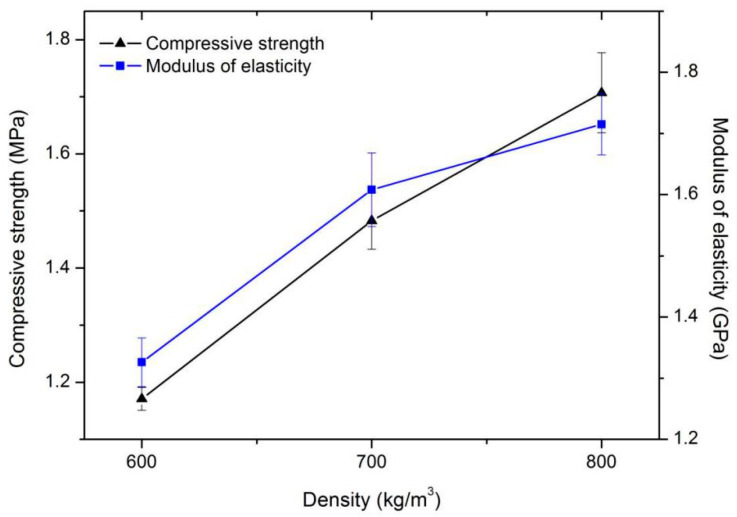
Compressive strength and modulus of elasticity of series I mixtures.

As shown in Figure 3, when the silt content was 20% and coarse particle content was 25%, the 28-day compressive strength and modulus of elasticity of foamed concrete showed the same characteristics and increased with the increase in wet density. For example, the compressive strength of foamed concrete increased by an average of 0.28 MPa along with a wet density increase of 100 kg/m^3^. This is because, as the density increased, the foam content in the mixture decreased and the content of cement that provided the strength increased. This pattern is similar to the results of foamed concrete mixed with clay obtained by Ma and Chen [25].

**Figure 4:**
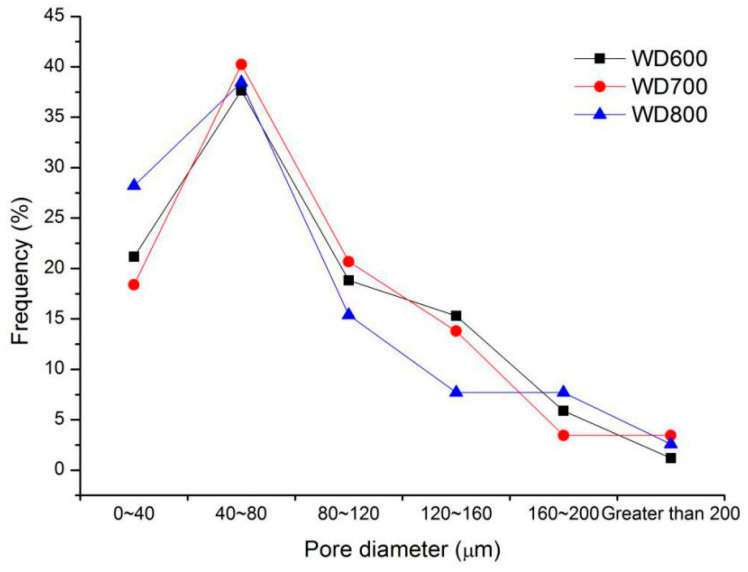
Pore size distribution of the air-void structure of series I mixtures.

The SEM results in Figure 4 show that the air-void structure was more compact with the increase in density, which show that the compressive strength and modulus of elasticity increased with wet density [26]. With the decrease in wet density, the air bubble in the foamed concrete increased; however, it also reduced the stability of the internal structure of foamed concrete [27], thus reducing its mechanical properties. The pore size distribution of the air-void structure was consistent in this density range, and about 40% of pores had a size between 40 µm and 80 µm.

### 3.2. Effect of Silt Content on the Properties of Foamed Concrete

**Figure 5:**
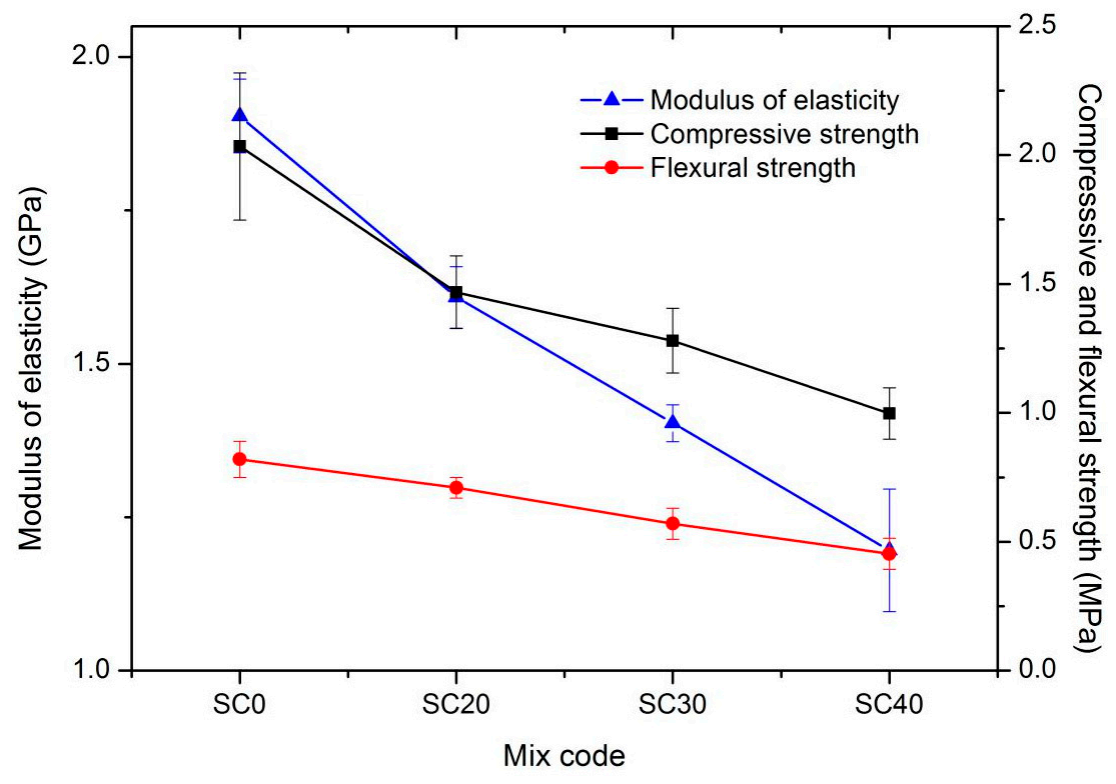
Mechanical properties of series II mixtures.

Figure 5 shows that, under the same wet density and coarse particle content, the compressive strength, flexural strength, and modulus of elasticity decreased with an increase in silt content. For every 10% increase in silt content, the compressive strength decreased by 21%, the flexural strength decreased by 12%, and the modulus of elasticity decreased by 14%. The main reason is that the cement plays the most important role in improving the strength of foamed concrete. However, for this new type of foamed concrete, because the cement was replaced by silt, the mechanical properties inevitably decreased with the increase in silt content.

**Figure 6:**
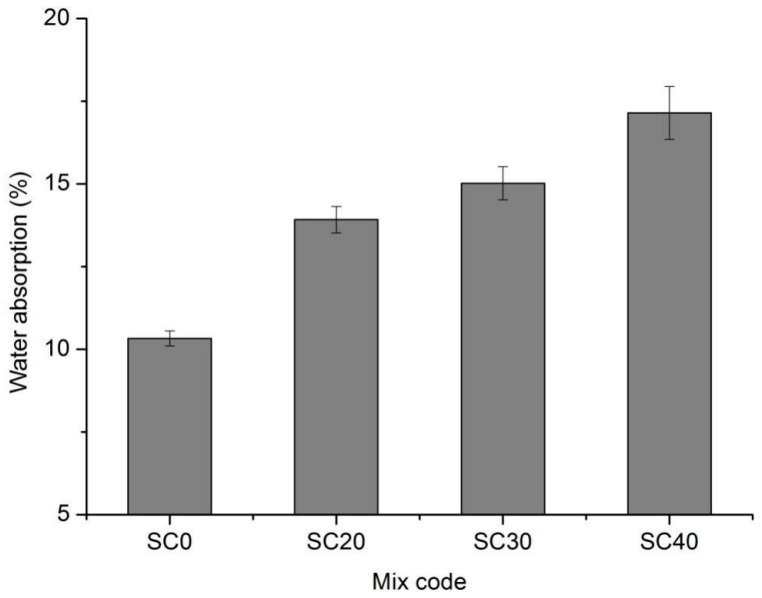
Water absorption of series II mixtures.

It can be seen from Figure 6 that the water absorption of foamed concrete increased significantly with the increase in silt content. With the increase in the proportion of silt, the amount of cement decreased, which means that the cementitious material decreased. Therefore, the foamed concrete slurry was thinner, and more capillary-size pores were formed after hardening. As a result, the intercommunicating pores that would be permeable inside the air-void structure increased, thus affecting the water absorption.

**Figure 7:**
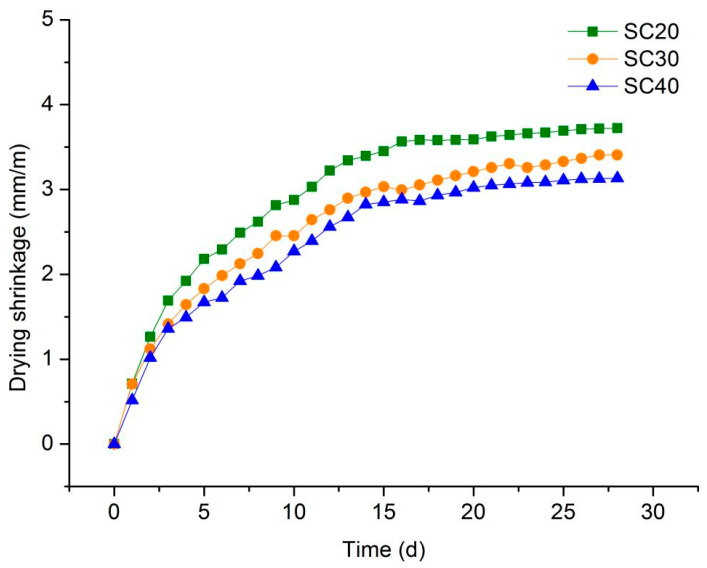
Drying shrinkage of series II mixtures.

Figure 7 shows that, with the increase in silt content, the drying shrinkage value of foamed concrete specimens gradually decreased. In other words, the decrease in cement content led to a decrease in drying shrinkage value. This was due to the solid-phase volume of ordinary Portland cement increasing during the hydration and hardening process, while the cement–water system shrunk. Secondly, the hydration process of cement was accompanied by a thermal effect which caused the initial volume to expand, and then the specimen shrunk as it cooled, resulting in an increase in the apparent shrinkage. Therefore, if the amount of cement was increased, the shrinkage of foamed concrete would increase accordingly. In a comparative study on the shrinkage behavior with sand and fly ash as filler, foam concrete with sand exhibited smaller drying shrinkage, which was attributed to the high shrinkage restraining capacity of sand [28]. In addition, silt is chemically inert, resulting in its inability to react with cement. After the addition of silt, this inertness of silt led to a significant reduction in the strength of foam concrete. However, due to this inertness, it had a “volume-invariable” property inside the foam concrete, which played the role of stabilizer. Therefore, the incorporation of silt is undoubtedly one of the measures able to reduce the drying shrinkage of foam concrete.

**Figure 8:**
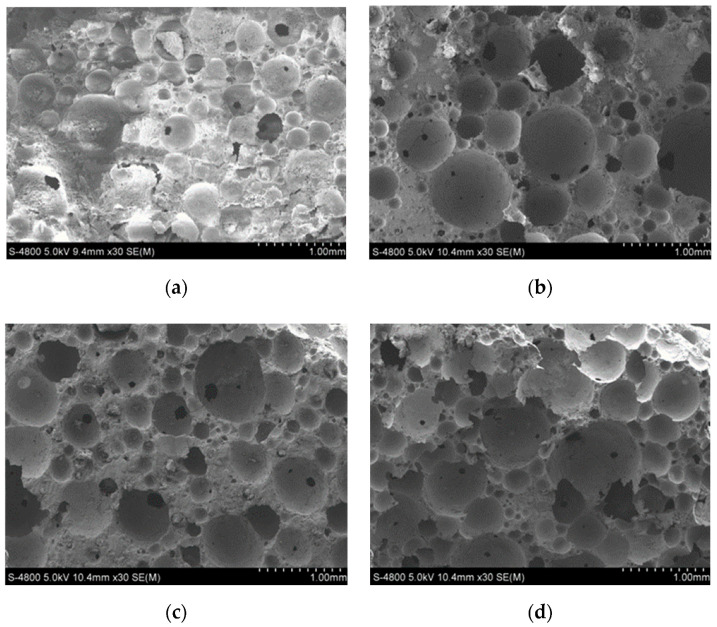
SEM images of pore structure of mixes with different silt content: (**a**) SC0; (**b**) SC20; (**c**) SC30; (**d**) SC40.

Figure 8 shows the microstructure pictures of four different contents of silt. It can be seen that the increase of silt content had a great impact on the air-void structure in the foamed concrete. When the amount of silt was low, the pores in the picture were more orderly, and there were few irregular bubbles. With the increase in silt content, the irregular air bubbles gradually increased, and the air-void structure began to break up. When the silt content reached 40%, the bubble-breaking group formed in the structure, as can be seen in the right corner of Figure 8d, which would have caused great damage to the mechanical structure inside the foamed concrete.

**Figure 9:**
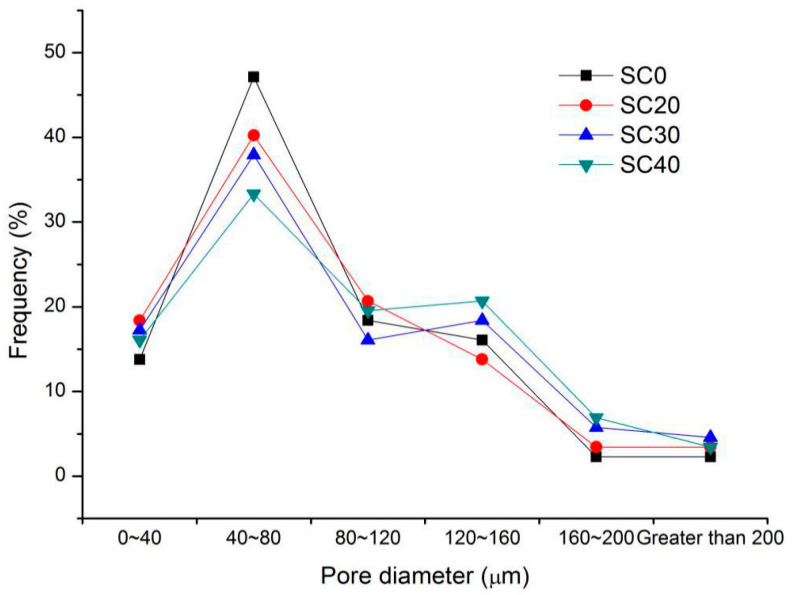
Pore size distribution of the air-void structure of series II mixtures.

It can be seen in Figure 9 that, with the increase in silt content, the bubbles with large pore size increased significantly. The uneven distribution of pores directly led to a decrease in strength and affected the mechanical properties [29]. Compared with cement particles, silt had a larger particle size and was relatively rough. When mixed with cement slurry, it caused certain damage to pores in the mixing process, thus affecting the microstructure after hardening. In addition, the increase in silt content meant that the amount of cementitious material was reduced. More foam and less cementitious material led to thinner pore walls [30], whereas the pores were even connected. At the same time, these factors made it easier for water to permeate the air-void structure of foam concrete and, thus, add to its water absorption.

### 3.3. Effect of Coarse Particle Content on the Properties of Foamed Concrete

**Figure 10:**
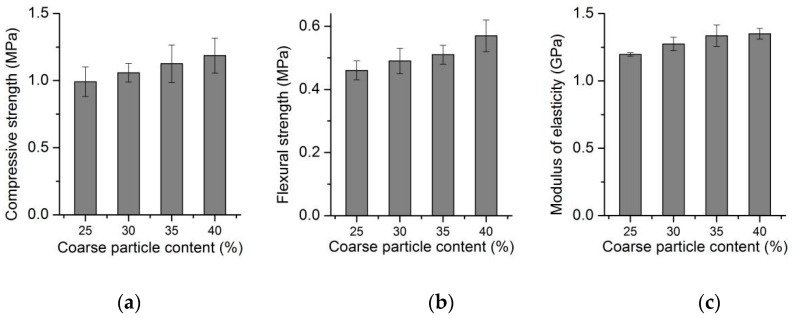
Mechanical properties of series III mixtures: (**a**) Effect of coarse particle content on compressive strength, (**b**) Effect of coarse particle content on flexural strength, (**c**) Effect of coarse particle content on modulus of elasticity.

Figure 10 shows that the compressive strength of foamed concrete increased gradually with the increase in coarse particle content under the same density and the same silt content. For every 5% increase in coarse particle content, the compressive strength increased by about 9–10%. When the coarse particle content in the silt increased from 25% to 40%, the flexural strength increased by 0.11 MPa. At the same time, the modulus of elasticity increased by an average of 0.05 GPa along with a 5% increase in coarse particle content.

Silt existed independently in foamed concrete and did not react with the cementitious system, which acted as a skeleton support inside the air-void structure, as well as a cheap filler for cementitious materials. With the increase in coarse particle content of silt, the mechanical properties of foamed concrete showed a tendency of gradual increase. On the one hand, when the silt particle size was prevalent, the particles were compact, which could better play the role of skeleton. On the other hand, when the coarse particle content of silt was prevalent, the water absorption and the viscosity of silt were relatively large. In the mixing process, too many fine particles made it easy to form flocculent and spherical accumulation. As a result, the air-void structure was destroyed, and the mechanical properties of foamed concrete were reduced.

**Figure 11:**
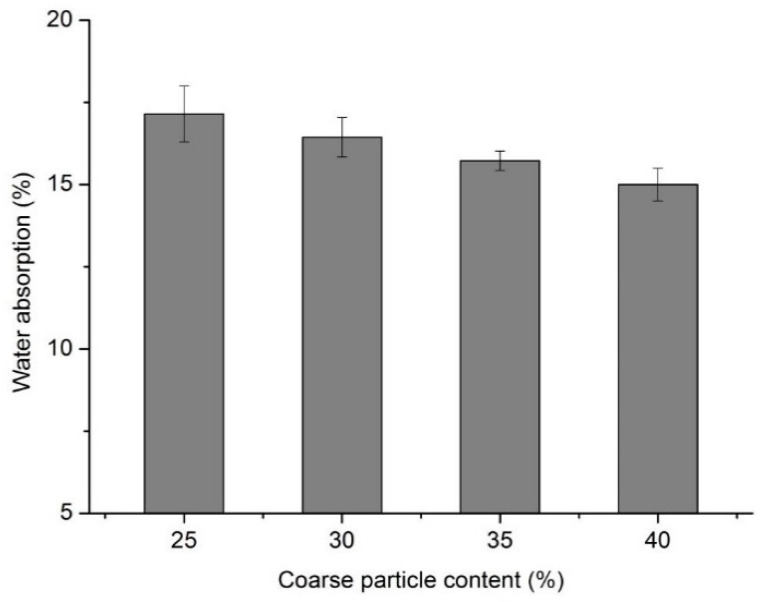
Water absorption of series III mixtures.

As can be seen from Figure 11, under the condition of the same silt content, a lower coarse particle content of silt led to a higher water absorption. When the content of coarse particles in silt was high, a relatively stable skeleton structure could be formed. A stable air-void structure was formed and led to a low water absorption. Compared with the coarse particles, the fine particles had higher water absorption, which further improved the water absorption of the foamed concrete.

**Figure 12:**
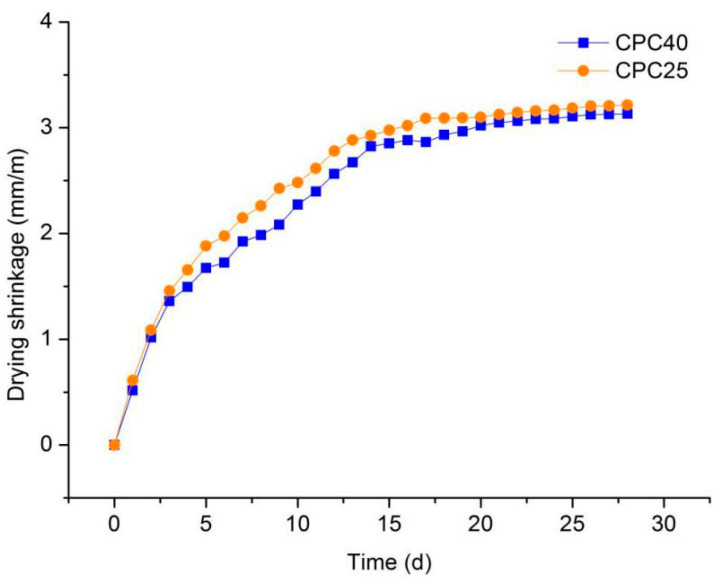
Drying shrinkage of series III mixtures.

Figure 12 compares the drying shrinkage of foamed concrete with two different silt coarse particle contents. It can be seen that a higher coarse grain content in the silt led to smaller drying shrinkage, because the coarse particles of silt were more stable than the fine particles. During the mixing of foamed concrete, the fine particles of the silt absorbed part of the water, which was stored in the foamed concrete. With the process of hydration in the foamed concrete, the stored water gradually escaped, resulting in a decrease in the specimen’s volume and an increase in its drying shrinkage.

**Figure 13:**
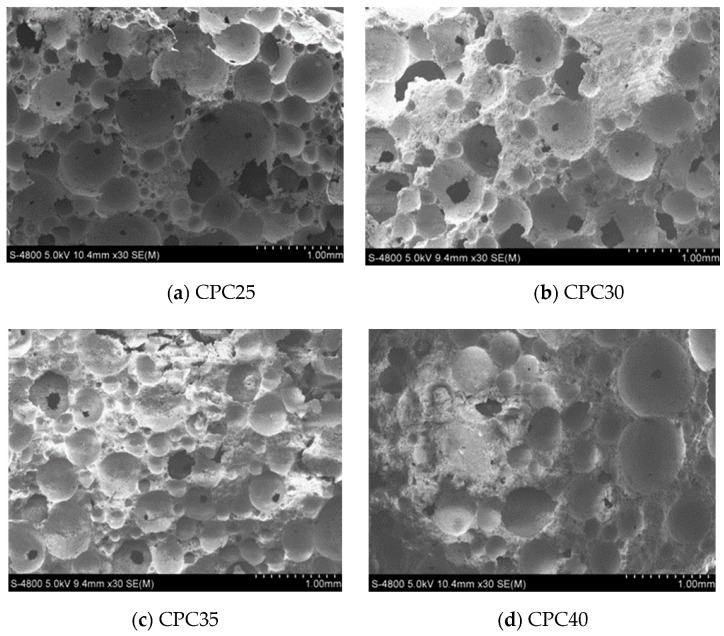
SEM images of pore structure of mixes with different coarse particle content: (**a**) CPC25; (**b**) CPC30; (**c**) CPC35; (**d**) CPC40.

Under the same density and silt content, upon comparing the four coarse particle contents in the SEM image, as seen in Figure 13a,b, there were many broken bubbles in the foamed concrete and the hole wall was very incomplete; thus, the mechanical properties were obviously reduced. When the content of coarse particles in silt was relatively high, the foamed concrete had a better and a relatively complete air-void structure, and there were almost no interconnected pores; thus, the mechanical properties of the concrete on the macro level were relatively high.

**Figure 14:**
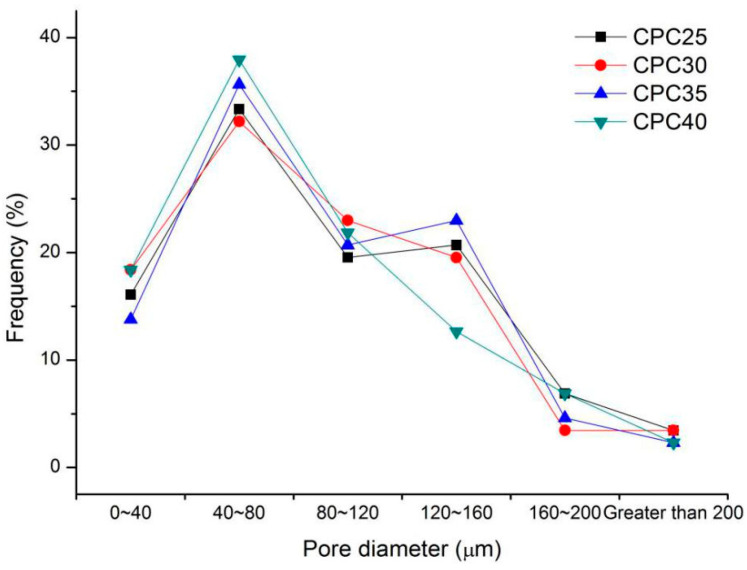
Pore size distribution of the air-void structure of series III mixtures.

According to Figure 14, with the increase in coarse particle content of silt, the distribution curve moved forward slightly, and the content of bubbles in the small pore size increased slightly. This could be because the increase in coarse particles broke up the bubbles in the large pore size during slurry mixing, such that the proportion of bubbles in the small pore size increased. In general, compared with other factors, the coarse particle content of silt was not the main factor affecting the microstructure of foamed concrete.

## 4. Conclusions and Discussion

Taking density, silt content, and coarse particle content into consideration, the mechanical and physical properties of silt-based foamed concrete were investigated. The experimental results of this investigation revealed the feasibility of widely applying silt-based foamed concrete, and the following conclusions can be drawn:

(1) High density, low silt content, or high coarse particle content led to better mechanical properties. The mechanical properties can all meet the requirements of the Chinese standard [22].
(2) Silt content was the main factor affecting the pore distribution of foamed concrete, and the addition of silt had an adverse effect on the air-void structure of foamed concrete. The change in air-void structure caused by the addition of silt correspondingly affected the water absorption rate.
(3) High silt content and high coarse particle content led to lower drying shrinkage. It is, thus, necessary to select an appropriate silt content according to the actual requirements.
(4) Considering the material performance and economy, it is recommended to use foamed concrete with a wet density of 700 kg/m^3^, silt content of 40%, and coarse particle content of 40%.

According to the investigation, higher coarse particle content would optimize the physical and mechanical properties of foamed concrete. However, this was only studied within the range of 25–40%. In a further investigation, silt with a wider range of coarse particle content needs to be added to the foamed concrete to explore whether there could be an inflection point in cases of the larger coarse grain content not being known.

## Figures and Tables

**Table 1:** Physical properties of the cement.

Blaine (m^2^/kg)	Normal Consistency (%)	Setting Time (min)		Compressive Strength (MPa)			Flexural Strength (MPa)		
		Initial Setting	Final Setting	3 Days	7 Days	28 Days	3 Days	7 Days	28 Days
358	27.9	215	270	28.1	31.9	50.7	6.5	7.8	9.6

**Table 2:** Chemical composition of cement.

Composition	CaO	SiO_2_	Al2O_3_	Fe2O_3_	MgO	SO_3_	The Total Amount of Alkali	Ignition Loss
Content (%)	64.63	21.96	4.73	3.68	2.59	0.3	0.63	2.89

**Table 3:** Parameters of raw soil.

Soil Type	d_10_ (mm)	d_30_ (mm)	d_60_ (mm)	C_u_	C_c_	Dry Density (cm^3^)	Water Content (%)
Silt	0.066	0.073	0.112	1.70	0.72	1.592	8.0
Clayey silt	0.004	0.013	0.023	5.75	1.84	1.487	8.1

**Table 4:** Parameters of different coarse particle contents.

Soil Type	d_10_ (mm)	d_30_ (mm)	d_60_ (mm)	C_u_	C_c_	Dry Density (cm^3^)	Water Content (%)
CPC-25%	0.047	0.121	0.530	11.28	0.58	1.541	8.1
CPC-30%	0.053	0.150	0.609	11.51	0.70	1.563	8.0
CPC-35%	0.058	0.202	0.691	11.90	1.00	1.598	8.0
CPC-40%	0.063	0.281	0.772	12.22	1.62	1.633	8.0

**Table 5:** Mixture compositions of foamed concrete. W/S—water-to-solid ratio.

Series	Mix Code	W/S	Target Wet Density (kg/m^3^)	Cement (kg/m^3^)	Water (kg/m^3^)	Foam (kg/m^3^)	Silt (kg/m^3^)	Wet Density (kg/m^3^)	Flow Value (mm)
I	WD600	0.55	600	292.72	201.27	32.84	73.17	585	17.5
	WD700	0.55	700	345.96	237.84	29.64	86.5	730	18.1
	WD800	0.55	800	399.16	274.41	26.64	99.79	793	18.6
II	SC0	0.55	700	431.46	237.31	31.23	0	704	16.7
	SC20	0.55	700	345.96	237.84	29.64	86.5	730	18.1
	SC30	0.55	700	303.09	238.14	28.87	129.89	718	19.0
	SC40	0.55	700	260.18	238.7	27.35	173.77	707	19.8
III	CPC25	0.55	700	260.18	238.7	27.35	173.77	707	18.8
	CPC30	0.55	700	260.09	238.42	28.14	173.39	731	19.2
	CPC35	0.55	700	260.04	238.37	28.2	173.37	695	19.7
	CPC40	0.55	700	260.2	238.24	28.25	173.3	728	19.9
